# Serum uric acid is associated with chronic kidney disease in elderly Chinese patients with diabetes

**DOI:** 10.1080/0886022X.2023.2238825

**Published:** 2023-07-24

**Authors:** Qing Zhou, Sisi Ke, Yaqiong Yan, Yan Guo, Qing Liu

**Affiliations:** a Department of Epidemiology, School of Public Health, Wuhan University, Wuhan, Hubei, P.R. China; b Wuhan Centers for Disease Control & Prevention, Wuhan, Hubei, P.R. China

**Keywords:** Serum uric acid, chronic kidney disease, elderly, diabetes

## Abstract

**Background:**

The relationship between hyperuricemia and chronic kidney disease (CKD) has been investigated extensively. However, studies on elderly individuals are still limited. Moreover, there is no consensus on whether hyperuricemia or elevated serum uric acid (SUA) within the normal range is correlated with the new onset of CKD and whether there are differences between males and females.

**Methods:**

We included 39039 elderly diabetic patients without CKD at baseline from a community-based cohort in Wuhan, China. The outcome event was the new onset of CKD (defined as an estimated glomerular filtration rate < 60 mL/min/1.73 m^2^). Multivariate Cox models were used to assess the adjusted hazard ratio (HR).

**Results:**

During the 2-year follow-up period, 3162 (8.10%) patients with diabetes developed new-onset CKD. The optimal cutoff value of SUA for incident CKD was 347.4 μmol/L. The adjusted HRs of hyperuricemia for new-onset CKD were 1.925 (1.724–2.150) and 1.676 (1.520–1.848) for males and females, respectively. The risk of developing CKD increased across the Q4 group up to 2.242 times for their counterparts in the lowest SUA quartile, independent of age, sex, diabetes duration, obesity, hypertension, systolic blood pressure, diastolic blood pressure, smoking, drinking, dyslipidemia, triglyceride, total cholesterol, high-density lipoprotein cholesterol, low-density lipoprotein cholesterol, and fasting plasma glucose.

**Conclusions:**

Hyperuricemia is an independent predictor of incident CKD. Elevated SUA was linearly correlated with CKD in elderly patients with diabetes, showing a relatively higher intensity among males compared with that among females. The optimal cutoff value of SUA for the risk of new-onset CKD in elderly patients with diabetes was 347.4 μmol/L.

## 1. Introduction

Diabetes is one of the most common chronic diseases worldwide and the leading cause of chronic kidney disease (CKD) in most countries. With the increasing ageing of the world’s population, the number of patients with diabetes is rapidly increasing, further increasing the incidence of CKD [1]. Indeed, approximately 40% of patients develop CKD [2], and this percentage even rises to 60% in Asians [3]. These patients have a poorer physical quality of life and a greater decrease in physical quality of life [4], which imposes a substantial social and economic burden [5].

In view of the fact that China is encountering the dual challenges of an aging population and the highest number of people with diabetes worldwide, it is particularly necessary to identify modifiable risk factors for CKD in elderly Chinese patients with diabetes. Increased serum uric acid (SUA) is not only an important risk factor for cardiovascular disease [6] and metabolic syndrome [7], but it has also been identified as a risk factor for CKD. An observational longitudinal study demonstrated that hyperuricemia seemed to be an independent risk factor for the development of incident CKD in patients with diabetes [8]. The association between higher SUA levels and a greater risk of CKD incidence was also found in the general population [9,10]. However, whether there is a linear, U-shaped [11] or J-shaped [12] association between SUA levels and CKD is still controversial. There is no consensus on this relationship between males and females  13,14].

Although epidemiologic studies have investigated the correlation between SUA and CKD, studies targeting the optimal cutoff value of SUA for CKD remain limited, especially in Chinese elderly adults with diabetes. In recent years, the popular research topic has shifted from the association between SUA and disease to the exploration of optimal cutoff values of SUA for different diseases, such as cardiovascular disease (CVD) incidence [15,16] and total and cardiovascular mortality (CVM) [17]. However, only one prospective cohort study analyzed the SUA cutoff for CKD progression [18].

Therefore, the primary focus of this community-based cohort study was to identify the optimal SUA threshold for incident CKD risk in Chinese adults with diabetes aged ≥ 65 years. We also examined whether elevated SUA levels, including those that are considered to be within the normal range, were an independent predictive factor for new-onset CKD in elderly adults with diabetes in China.

## 2. Materials and methods

### 2.1. Study design and participants

**Figure 1.**
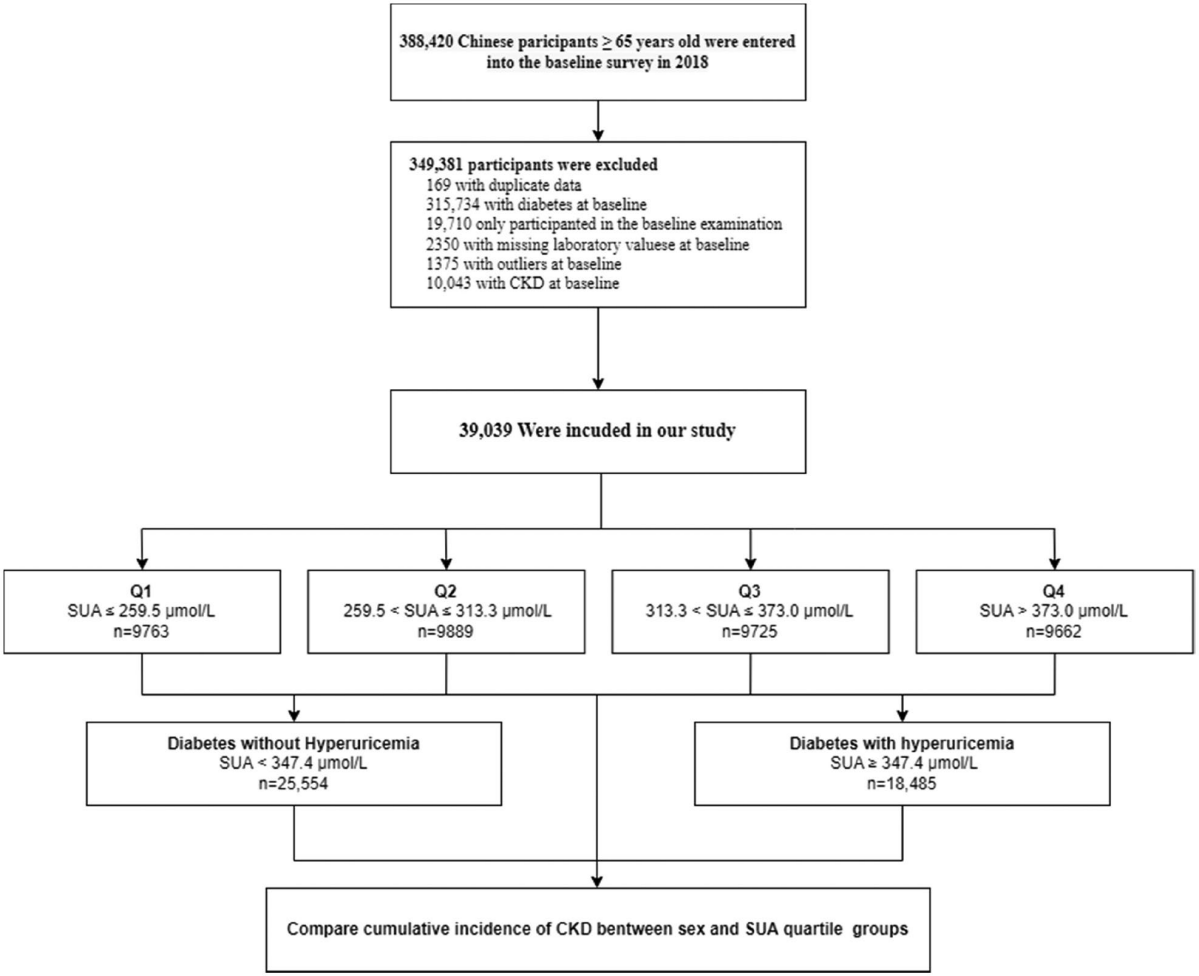
Flowchart of study participants.

Our study was based on geriatric health services, a vital component of the National Basic Public Health services in China [19]. Community health service center staff recruited elderly people aged ≥65 years in the district to participate in free questionnaires, physical examinations and laboratory tests. We constructed a cohort of patients with diabetes from these subjects and then divided them into four groups based on SUA quartiles. Those with incomplete data, including demographics, lifestyle, anthropometric indices and laboratory examinations, were excluded. Participants who only participated in the baseline examination were also excluded from the study (Figure 1). The study protocol was reviewed and approved by the Ethics Committee of Wuhan Centers for Disease Control and Prevention (#WHCDCIRB-K-2018023).

### 2.2. Data collection

The standard health status questionnaire was used to obtain basic demographic information, such as age, sex, diabetes duration, and lifestyle factors, including smoking and drinking status. Smoking status was defined as having smoked cigarettes more than once a month [20]. Drinking status was defined as drinking alcohol once or more per month [21]. Anthropometric examinations of each participant were performed by professional community doctors. Height and weight measurements were collected with the subjects dressed in lightweight clothes and no shoes. The body mass index (BMI) was calculated using the following equation: BMI = weight (kg)/height (m^2^). Blood pressure was measured three times using an electronic sphygmomanometer with participants in a sitting position, and the mean of the three measurements was used for subsequent analyses. After an overnight fasting period (at least 8 h), blood samples were collected from participants for laboratory tests.

### 2.3. Definition

Participants were considered to have reached the outcome of CKD when the estimated glomerular filtration rate (eGFR) was <60 mL/min/1.73 m^2^ [22]. Additionally, the eGFR was evaluated using the formula eGFR (mL/min/1.73 m^2^) = 175 × serum creatinine (Scr) (μmol/L)^−1.24^ × age (year)^−0.179^ [female × 0.79] [23]. CKD stages were categorized as follows: G1 (eGFR ≥ 90 mL/min/1.73 m^2^) and G2 (60–89 mL/min/1.73 m^2^) [24].

In accordance with the diagnostic criteria from the American Diabetes Association [25], diabetes was defined as fasting plasma glucose (FPG) ≥7.0 mmol/L or self-reported diagnosis by professional physicians. Obesity was defined as BMI ≥24 kg/m^2^. Hypertension was defined as systolic blood pressure (SBP) ≥140 mmHg, diastolic blood pressure (DBP) ≥90 mmHg, self-reported preceding diagnosis by specialists, or self-reported taking of anti-hypertension medication [26]. According to the 2016 Chinese guidelines for the management of dyslipidemia in adults [27], dyslipidemia was defined as TC ≥6.2 mmol/L and/or TGs ≥2.3 mmol/mol and/or HDL-*C* < 1.0 mmol/L and/or LDL-*C* ≥ 4.1 mmol/L.

The participants were considered to have hyperuricemia when the SUA level was ≥347.4 μmol/L, which was assessed by a time-dependent receiver operating characteristic (ROC) curve. We then divided diabetes into four groups based on SUA quartile as follows: Q1 (≤259.5 μmol/L), Q2 (259.5–313.3 μmol/L), Q3 (313.3–373.0 μmol/L) and Q4 (>373 μmol/L).

### 2.4. Statistical analysis

The data were double-entered by two trained data managers using EpiData (version 3.2; The EpiData Association, Odense, Denmark). Normally distributed and continuous variables were expressed as the mean ± standard deviation. Nonnormally distributed data are presented as the median (interquartile range). Categorical variables are presented as percentages. Baseline characteristics of the different groups were analyzed by ANOVA (for continuous variables) or Pearson’s chi-square (for categorical variables) tests. Cox regression analysis was performed to estimate the association between SUA levels and incident CKD. The optimal cutoff of SUA was identified by time-dependent ROC analysis to find the maximum value of the Youden index. Propensity score matching was performed to balance the covariance of the participants. The balance of covariance between the two groups based on the presence of hyperuricemia was assessed using the standardized mean difference (SMD), with SMD <0.1 indicating a balance characteristic between the two groups. The results are presented as hazard ratios (HRs) and 95% confidence intervals (CI). Statistical significance was set at *p* < 0.05. The data were analyzed using SAS for Windows (version 9.4; SAS Institute, Cary, NC, United States).

## 3. Results

### 3.1. Baseline characteristics of participants

By the study design, a total of 39,039 elderly patients with diabetes were included in this study after fulfilling the inclusion criteria. Complete information on demographics, anthropometric indices, lifestyles, and laboratory examinations was obtained from all subjects. Overall, the mean age of the cohort was 71.91 ± 5.09 years. The average SUA level was 320.78 ± 88.80 μmol/L. Baseline statistics were 72.53 ± 16.36 μmol/L for Scr and 92.01 (76.85–111.35) mL/min/1.73 m^2^ for eGFR.

The demographic and clinical characteristics of the subjects grouped according to the presence of hyperuricemia are summarized in Table 1. Compared with patients without hyperuricemia, individuals with hyperuricemia were more likely to be older, male, and have higher Scr and lower baseline eGFR. The prevalence of obesity, hypertension, and dyslipidemia was markedly higher in the hyperuricemia group.

**Table 1.** Characteristics of patients with diabetes according to the presence of hyperuricemia.

	Patients with diabetes		
Characteristic	Without hyperuricemia (*N* = 25,554)	Hyperuricemia (*N* = 13,485)	*P* value
Age	71.83 ± 5.03	72.07 ± 5.19	<0.001
Sex (female *n*, %)	16617 (65.03%)	6271 (46.50%)	<0.001
Diabetes duration	5.0(0-10.0)	4.0(0–10.0)	<0.001
BMI (kg/m^2^)	24.41 ± 3.23	25.40 ± 3.21	<0.001
Obesity (*n*, %)	13,531 (52.95%)	8857(65.68%)	<0.001
Hypertension (*n*, %)	19,485 (76.25%)	10,967 (81.33%)	<0.001
SBP (mmHg)	138.33 ± 18.95	138.56 ± 18.42	0.247
DBP (mmHg)	78.97 ± 10.62	79.55 ± 10.76	<0.001
Smoking (*n*, %)	2788(10.91%)	1948 (14.45%)	<0.001
Drinking (*n*, %)	2861 (11.20%)	2479 (18.38%)	<0.001
Dyslipidemia (*n*, %)	8032 (31.43%)	5666(42.02%)	<0.001
TGs (mmol/L)	1.29(0.93-1.82)	1.55(1.10-2.22)	<0.001
TC (mmol/L)	4.81 ± 1.05	4.80 ± 1.08	0.427
HDL-C (mmol/L)	1.36 ± 0.36	1.26 ± 0.33	<0.001
LDL-C (mmol/L)	2.63 ± 0.87	2.63 ± 0.88	0.693
FPG ((mmol/L)	7.86 ± 2.91	7.43 ± 2.45	<0.001
Scr (μmol/L)	69.54 ± 15.69	78.21 ± 16.09	<0.001
eGFR categories			<0.001
G1	15,147(59.27%)	5644(41.85%)	
G2	10,407(40.73%)	7841(58.15%)	

Data are shown as the mean ± standard deviation for normally distributed variables, median (interquartile range) for nonnormally distributed variables, or percentages for categorical variables.

BMI: body mass index; SBP: systolic blood pressure; DBP: diastolic blood pressure; TGs: triglyceride; TC: total cholesterol; HDL-C: high-density lipoprotein cholesterol; LDL-C: low-density lipoprotein cholesterol; FPG: fasting plasma glucose; Scr: serum creatinine; eGFR: estimated glomerular filtration rate. SBP, DBP, TGs, TC, HDL-C, and LDL-C levels were included as continuous variables.

### 3.2. SUA threshold for CKD

**Figure 2.**
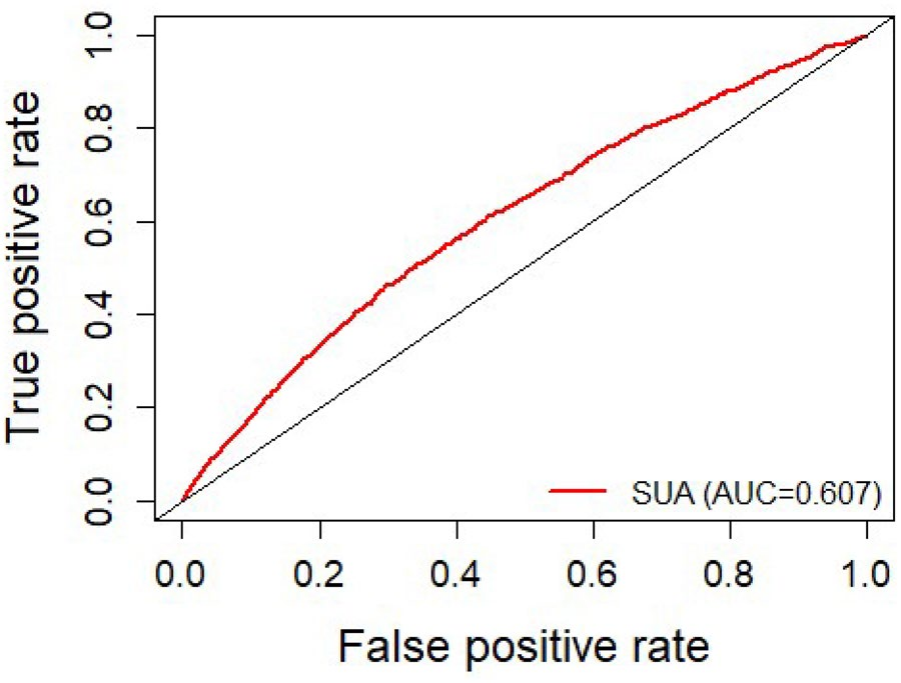
Receiver operating characteristic curve to evaluate the predictive capacity of serum uric acid on 2-year chronic kidney disease incidence through the area under the curve in patients with diabetes.

**Figure 3.**
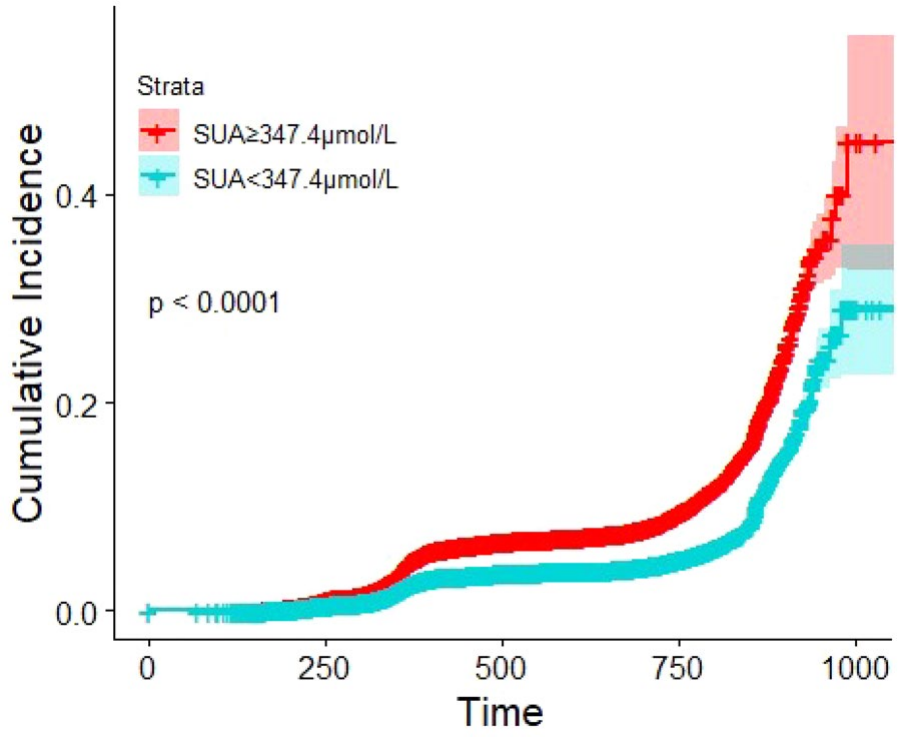
Kaplan–Meier survival estimates according to the identified cutoff for risk of CKD (*p* < 0.0001, log-rank test); analysis time is expressed in days. CKD: chronic kidney disease; SUA: serum uric acid.

The survival ROC curve analysis is presented in Figure 2 and shows that the optimal cutoff value of SUA for predicting 2-year CKD incidence was 347.4 μmol/L. SUA levels ≥347.4 μmol/L were correlated with an increased risk of new-onset CKD in elderly diabetes patients (Figure 3).

### 3.3. Relationships of hyperuricemia and SUA levels with the new onset of CKD

The relationships between hyperuricemia and SUA levels with the new onset of CKD in diabetes are displayed in Table 2. During the 2-year follow-up period, 3162 patients with diabetes developed new-onset CKD. The unadjusted incidence of CKD in the study population was 8.10% (95% CI 7.83, 8.37).

**Table 2.** The Graded association between serum uric acid (SUA) and new onset of CKD in participants with diabetes before PSM and after PSM.

SUA quartiles	Model 1		Model 2	
	Adjusted HR (95% CI)	*P* value for trend	Adjusted HR (95% CI)	*P* value for trend
Unmatched sample				
Normal HUA	1.000		1.000	
Hyperuricemia	1.819 (1.694–1.953)		1.778 (1.653–1.912)	
SUA(categorical)		<0.001		<0.001
Q1	1.000		1.000	
Q2	1.191 (1.063–1.335)		1.168 (1.042–1.309)	
Q3	1.522 (1.364–1.698)		1.486 (1.330–1.660)	
Q4	2.319 (2.091–2.573)		2.242 (2.016–2.494)	
Matched				
Normal HUA	1.000		1.000	
Hyperuricemia	1.743 (1.606–1.893)		1.750 (1.612–1.900)	

Hazard ratio (HR) and 95% confidence interval (95% CI) for new-onset CKD.

Model 1: adjusted for age and sex.

Model 2: adjusted for age, sex, diabetes duration, obesity, hypertension, SBP, DBP, smoking, drinking, dyslipidemia, TGs, TC, HDL-C, LDL-C, and FPG.

CKD: chronic kidney disease; PSM: propensity score matching; SBP: systolic blood pressure; DBP: diastolic blood pressure; TGs: triglyceride; TC: total cholesterol; HDL-C, high-density lipoprotein cholesterol; LDL-C, low-density lipoprotein cholesterol; FPG, fasting plasma glucose.

SBP, DBP, TGs, TC, HDL-C, LDL-C, and FPG levels were included as dichotomous variables. SBP was defined as SBP ≥ 140 mmHg. DBP was defined as DBP ≥ 90 mmHg. TGs were defined as TGs ≥2.3 mmol/L. TC was defined as TC ≥6.2 mmol/L. HDL-C was defined as HDL-*C* ≤ 1.0 mmol/L. The LDL-C level was defined as LDL-*C* ≥ 4.1 mmol/L. FPG was defined as FPG ≥7.0 mmol/L.

Cox regression analysis indicated that compared to the subjects in Q1, the risk of developing CKD gradually and linearly increased across SUA levels. After multivariate adjustment, the risk of CKD in Q4 was 2.242-fold greater than that of the reference group.

Similar analyses were conducted when hyperuricemia was defined with the new cutoff value. Cox regression analysis demonstrated that incident CKD was independently associated with hyperuricemia (*P* value <0.001). The risk of developing new-onset CKD increased across the hyperuricemia group by up to 1.819 times for their counterparts without hyperuricemia when we only adjusted for age and sex. After further adjustment for multiple potential confounders, this correlation was attenuated but remained statistically significant.

Propensity score matching (PSM) was performed to balance the covariances of participants between the hyperuricemia group and the normal SUA group. Table S1 shows that the covariances were well-balanced for all SMDs <0.1. Hyperuricemia was associated with new onset of CKD after PSM (1.750 95% CI 1.612–1.900).

### 3.4. Determinants of the new onset of CKD among sex-specific groups

Univariate and multivariate Cox regression analyses for variables independently associated with new-onset CKD are presented in Tables 3 and 4. Factors correlated with the new onset of CKD were age, diabetes duration, obesity, hypertension, SBP, DBP, smoking, drinking, dyslipidemia, TGs, HDL-C, and hyperuricemia among males based on univariate Cox regression. In the multivariate Cox regression analysis, variables including obesity, SBP, DBP, smoking, dyslipidemia, TGs and HDL-C were removed from the model. Only drinking was a protective factor, while other factors were independent risk factors for males. The PSM analysis, after balancing the covariates of participants, yielded similar results.

**Table 3.** Univariate and multivariate Cox regression for independently associated variables with the new onset of CKD in males.

Variables	Unmatched (*n* = 16,151)					Matched (*N* = 14,254)		
	Rate (%)	Crude HR (95% CI)	*P*	Adjusted HR (95% CI)	*P*	Rate (%)	Adjusted HR (95% CI)	*P*
Age								
65–74	72.97	1.000		1.000		72.37	1.000	
75–84	24.60	1.380 (1.227–1.551)	<0.001	1.344 (1.193–1.514)	<0.001	25.12	1.354 (1.197–1.532)	<0.001
≥85	2.43	2.301 (1.775–2.982)	<0.001	2.112 (1.623–2.749)	<0.001	2.51	2.113 (1.612–2.770)	<0.001
Diabetes duration								
<5	53.75	1.000		1.000		54.29	1.000	
5–10	25.42	1.211 (1.066–1.375)	0.003	1.187 (1.043–1.351)	0.009	25.59	1.203 (1.053–1.375)	0.007
>10	20.83	1.356 (1.188–1.547)	<0.001	1.404 (1.228–1.606)	<0.001	20.11	1.404 (1.220–1.616)	<0.001
Obesity								
No	43.17	1.000		1.000		37.55	1.000	
Yes	56.83	1.221 (1.095–1.361)	<0.001	1.099(0.982–1.230)	0.099	62.45	1.093 (0.972–1.229)	0.139
Hypertension								
No	23.68	1.000		1.000		21.21	1.000	
Yes	76.32	1.574 (1.364–1.817)	<0.001	1.358(1.151–1.601)	<0.001	78.79	1.353 (1.137–1.609)	<0.001
SBP								
No	52.36	1.000		1.000		51.84	1.000	
Yes	47.64	1.227 (1.103–1.364)	<0.001	1.041 (0.917–1.181)	0.538	48.16	1.035 (0.909–1.179)	0.603
DBP								
No	78.07	1.000		1.000		77.80	1.000	
Yes	21.93	1.167 (1.033–1.319)	0.013	1.118 (0.978–1.279)	0.103	22.20	1.143 (0.996–1.313)	0.058
Smoking								
No	72.60	1.000		1.000		73.73	1.000	
Yes	27.40	0.882 (0.782–0.996)	0.044	1.045 (0.921–1.186)	0.494	26.27	1.098 (0.963–1.253)	0.163
Drinking								
No	69.74	1.000		1.000		68.32	1.000	
Yes	30.26	0.789 (0.698–0.891)	<0.001	0.786 (0.692–0.892)	<0.001	31.68	0.779 (0.684–0.887)	<0.001
Dyslipidemia								
No	65.92	1.000		1.000		63.06	1.000	
Yes	34.08	1.237 (1.109–1.378)	<0.001	1.149 (0.888–1.485)	0.291	36.94	1.129 (0.869–1.465)	0.363
TGs								
No	86.42	1.000		1.000		84.61	1.000	
Yes	13.58	1.261 (1.092–1.456)	0.002	1.076 (0.875–1.322)	0.488	15.39	1.080 (0.876–1.331)	0.473
TC								
No	94.38	1.000		1.000		94.04	1.000	
Yes	5.62	1.165 (0.944–1.438)	0.155	1.064 (0.812–1.394)	0.655	5.96	1.045 (0.793–1.377)	0.756
HDL-C								
No	79.42	1.000		1.000		77.75	1.000	
Yes	20.58	1.150 (1.014–1.304)	0.030	0.961 (0.759–1.216)	0.739	22.25	0.959 (0.756–0.215)	0.727
LDL-C								
No	96.15	1.000		1.000		95.99	1.000	
Yes	3.85	1.136 (0.883–1.460)	0.321	0.974 (0.716–1.325)	0.867	4.01	0.992 (0.724–1.360)	0.960
FPG								
No	39.47	1.000		1.000		41.67	1.000	
Yes	60.53	0.910 (0.817–1.013)	0.085	0.971 (0.868–1.086)	0.604	58.33	0.972 (0.865–1.091)	0.626
Hyperuricemia								
No	55.33	1.000		1.000		49.39	1.000	
Yes	44.67	1.985 (1.781–2.213)	<0.001	1.925 (1.724–2.150)	<0.001	50.61	1.886 (1.679–2.117)	<0.001

Hazard ratios (HRs) and 95% confidence intervals (95% CIs) for new-onset CKD.

Adjusted for age, diabetes duration, obesity, hypertension, SBP, DBP, smoking, drinking, dyslipidemia, TGs, TC, HDL-C, LDL-C, FPG, and hyperuricemia.

CKD: chronic kidney disease; SBP: systolic blood pressure; DBP: diastolic blood pressure; TGs: triglyceride; TC: total cholesterol; HDL-C, high-density lipoprotein cholesterol; LDL-C, low-density lipoprotein cholesterol; FPG, fasting plasma glucose.

SBP, DBP, TGs, TC, HDL-C, LDL-C, and FPG levels were included as dichotomous variables. SBP was defined as SBP ≥140 mmHg. DBP was defined as DBP ≥90 mmHg. TGs were defined as TGs ≥ 2.3 mmol/L. TC was defined as TC ≥ 6.2 mmol/L. HDL-C was defined as HDL-*C* ≤ 1.0 mmol/L. The LDL-C level was defined as LDL-*C* ≥ 4.1 mmol/L. FPG was defined as FPG ≥7.0 mmol/L.

**Table 4.** Univariate and multivariate Cox regression for independently associated variables with the new onset of CKD in females.

Variables	Unmatched (*n* = 22,888)					Matched (*N* = 12,716)		
	Rate (%)	Crude HR (95% CI)	*P*	Adjusted HR (95% CI)	*P*	Rate (%)	Adjusted HR (95% CI)	*P*
Age								
65–74	73.89	1.000		1.000		72.55	1.000	
75–84	24.14	1.775 (1.607–1.961)	<0.001	1.728 (1.563–1.912)	<0.001	25.50	1.706 (1.507–1.931)	<0.001
≥85	1.97	3.836 (3.105–4.741)	<0.001	3.752 (3.031–4.644)	<0.001	1.96	3.216 (2.427–4.263)	<0.001
Diabetes duration								
<5	48.63	1.000		1.000		49.92	1.000	
5–10	29.19	1.112 (0.998–1.239)	0.055	1.126 (1.010–1.257)	0.033	29.50	1.164 (1.017–1.331)	0.023
>10	22.18	1.206 (1.072–1.356)	0.002	1.192 (1.058–1.342)	0.004	20.58	1.239 (0.968–1.316)	0.122
Obesity								
No	42.29	1.000		1.000		31.74	1.000	
Yes	57.71	1.153 (1.048–1.268)	0.003	1.072 (0.9731–.182)	0.160	68.26	1.028 (0.905–.167)	0.672
Hypertension								
No	20.81	1.000		1.000		16.36	1.000	
Yes	79.19	1.364 (1.202–1.548)	<0.001	1.071 (0.925–1.243)	0.353	83.64	1.084 (0.892–1.318)	0.418
SBP								
No	50.97	1.000		1.000		49.98	1.000	
Yes	49.03	1.276 (1.163–1.401)	<0.001	1.156 (1.034–1.292)	0.011	50.02	1.212 (1.058–1.388)	0.006
DBP								
No	82.33	1.000		1.000		81.57	1.000	
Yes	17.67	1.231 (1.10–11.376)	<0.001	1.196 (1.061–1.349)	0.003	18.43	1.207 (1.042–1.398)	0.012
Smoking								
No	98.64	1.000		1.000		98.80	1.000	
Yes	1.36	1.226 (0.849–1.769)	0.277	1.274 (0.878–1.849)	0.203	1.20	1.505 (0.959–2.363)	0.076
Drinking								
No	98.03	1.000		1.000		97.88	1.000	
Yes	1.97	0.854 (0.599–1.218)	0.383	0.777 (0.542–1.114)	0.169	2.12	0.605 (0.376–0.972)	0.034
Dyslipidemia								
No	64.20	1.000		1.000		54.71	1.000	
Yes	35.80	1.142 (1.038–1.255)	0.006	1.062 (0.863–1.307)	0.570	45.29	1.015 (0.794–1.297)	0.908
TGs								
No	80.14	1.000		1.000		70.29	1.000	
Yes	19.86	1.154 (1.031–1.291)	0.013	1.015 (0.853–1.208)	0.867	29.71	0.999 (0.815–1.225)	0.994
TC								
No	88.02	1.000		1.000		86.51	1.000	
Yes	11.98	1.158 (1.014–1.324)	0.031	1.095 (0.906–1.323)	0.349	13.49	1.098 (0.881–1.369)	0.404
HDL-C								
No	88.27	1.000		1.000		85.03	1.000	
Yes	11.73	1.049 (0.912–1.206)	0.502	0.953 (0.786–1.155)	0.621	14.97	1.031 (0.834–1.274)	0.780
LDL-C								
No	93.52	1.000		1.000		93.29	1.000	
Yes	6.48	1.074 (0.900–1.283)	0.428	0.966 (0.778–1.201)	0.758	6.71	0.837 (0.633–1.108)	0.215
FPG								
No	43.73	1.000		1.000		46.16	1.000	
Yes	56.27	0.974 (0.887–1.069)	0.579	0.987 (0.897–1.087)	0.796	53.84	0.956 (0.848–1.077)	0.456
Hyperuricemia								
No	72.60	1.000		1.000		50.68	1.000	
Yes	27.40	1.746 (1.587–1.920)	<0.001	1.676 (1.520–1.848)	<0.001	49.32	1.609 (1.430–1.811)	<0.001

Hazard ratios (HRs) and 95% confidence intervals (95% CIs) for new-onset CKD.

Adjusted for age, diabetes duration, obesity, hypertension, SBP, DBP, smoking, drinking, dyslipidemia, TGs, TC, HDL-C, LDL-C, FPG, and hyperuricemia.

CKD: chronic kidney disease; SBP: systolic blood pressure; DBP: diastolic blood pressure; TGs: triglyceride; TC: total cholesterol; HDL-C, high-density lipoprotein cholesterol; LDL-C, low-density lipoprotein cholesterol; FPG, fasting plasma glucose.

SBP, DBP, TGs, TC, HDL-C, LDL-C, and FPG levels were included as dichotomous variables. SBP was defined as SBP ≥ 140 mmHg. DBP was defined as DBP ≥ 90 mmHg. TGs was defined as TGs ≥ 2.3 mmol/L. TC was defined as TC ≥ 6.2 mmol/L. HDL-C was defined as HDL-*C* ≤ 1.0 mmol/L. The LDL-C level was defined as LDL-*C* ≥ 4.1 mmol/L. FPG was defined as FPG ≥ 7.0 mmol/L.

However, in females, these associations were slightly different. Univariate analysis demonstrated that smoking, drinking and lower HDL-C were related factors for new-onset CKD in males but not in females. The relationships between incident CKD and SBP or DBP were attenuated but remained statistically significant in females after multivariable adjustment. The associations between the new onset of CKD and hypertension and higher TGs completely disappeared in the adjusted model. However, in PSM analysis, drinking was found to be a correlated factor for new onset of CKD in females. The adjusted HR for hyperuricemia in males (1.925, 95% CI 1.724–2.150) tended to be slightly higher than that in females (1.676, 95% CI 1.520–1.848).

## 4. Discussion

In this community-based cohort study, we found that in parallel with increasing baseline SUA levels, the risk of developing CKD in diabetes linearly increased. The optimal cutoff value of SUA for incident CKD was 347.4 μmol/L. Based on this cutoff, hyperuricemia was considered an independent risk factor for incident CKD, with a stronger association in males than in females. Our findings suggested that strategies for stringent SUA control were likely to reduce the risk of new onset of CKD in diabetes.

The present study identified that the cutoff of SUA on incident CKD in older Chinese diabetes suggested by ROC analysis was 347.4 μmol/L, which was 17.29% lower than the value routinely used for the diagnosis of hyperuricemia. Similar results have been reported in previous studies. The cutoff value of SUA for detecting the 10-year CVD event rate was 4.15 mg/dL (249 μmol/L) in women and 5.05 mg/dL (303 μmol/L) in men [15]. The SUA threshold was 4.7 mg/dL (282 μmol/L) in discriminating total mortality and 5.6 mg/dL (336 μmol/L) in discriminating CVM status [17]. This evidence suggests that the risk of morbidity or mortality may be increased even at lower SUA levels. Our findings show that it is necessary to reset the cutoff value of SUA and determine the threshold according to specific elderly populations.

The association that was found in this study supported the findings of other published studies [8,28], but it is worth noting that the linear correlation was different from the previously reported U-shaped association [11,29] or J-shaped association [12]. A study based on health checkups in the elderly population reported results that were similar to those in our study [14]. Therefore, this difference may be partly explained by the age of the study subjects, as the people in those other studies were middle-aged individuals, whereas the subjects in our study were elderly individuals. An association between baseline SUA levels and subsequent incident CKD was detected in the SUA quartiles. This association persisted after adjusting for potential confounding factors and remained statistically significant, even in the normal range of SUA levels.

The discovery of SUA as an independent risk factor for the new onset of CKD does not imply causation, but many studies have demonstrated that SUA plays an important role in the pathogenesis of diabetic kidney disease. The putative underlying mechanisms can be summarized as impaired nitric oxide generation, chronic inflammation, endothelial dysfunction and oxidative stress [30–33]. The levels of fructose in serum and urine were higher in individuals with diabetes than in individuals without diabetes [34]. Intracellular fructose activated by the polyol pathway can be further metabolized to generate uric acid as a side product [35]. The generated uric acid further promotes renal function damage through the above mechanisms.

In the present study, we noted that the correlation between alcohol consumption and new onset of CKD was negative. The common knowledge that alcohol consumption causes liver [36] and neurological diseases [37] may lead to the hasty assumption that drinking is also a risk factor for CKD. However, this relationship is inconclusive. Population-based cohort studies corroborated that alcohol consumption was inversely associated with the risk of new-onset CKD [38,39], especially in elderly males [40]. A possible mechanism for this correlation is that the polyphenolic compounds in alcohol have an antioxidant effect. Resveratrol, a polyphenolic compound, has been reported to reduce the level of serum creatinine in patients with diabetes [41], which suggests that drinking improves kidney function.

A separate significant finding worth noting was that the association between hyperuricemia and the new onset of CKD in males was relatively stronger than that in females, with the adjusted HR of hyperuricemia for CKD being 1.925 (1.724–2.150) and 1.676 (1.530–1.848) for males and females, respectively. Previous studies have investigated sex differences in the correlations between hyperuricemia and incident CKD but obtained mixed results [13,42,43]. Estrogen is the most commonly considered reason for this sex difference, as it can cause the excretion of urate through urine [44] and inhibit the generation of uric acid by xanthine oxidase [45]. However, all elderly females in our study were postmenopausal, which diminished the confidence in this interpretation. We speculated that residual estrogen still plays a protective role and that there may be other potential risk factors in elderly males.

Although there is currently no consensus on the relationship between SUA levels and CKD [46], animal experimental evidence supports our viewpoint through several pathogenic mechanisms. When SUA levels increase and further develop into hyperuricemia, endothelial dysfunction occurs by inhibiting the production of nitric oxide [47], which plays an important role in the incidence of CKD [48]. Increased activity of the renin-angiotensin aldosterone system by SUA may also exert effects [49].

This study has some limitations. First, considering that the presence of albuminuria was not included in the definition of CKD, incident CKD might have been underestimated in this study. Second, we used eGFR calculated using the simplified Chinese Modification of Diet in Renal Disease (MDRD) equation instead of a directly measured GFR to define CKD, which may result in underestimation of GFR and lead to overdiagnosis of CKD. However, it is infeasible to directly measure GFR during free physical examinations because of its complexity. Meanwhile, we lack information on the adherence to diet and therapy of patients with hyperuricemia. Moreover, our findings should be generalized to the entire geriatric population with caution since only patients who could be followed-up were included in the analysis.

In conclusion, the optimal cutoff value of SUA for the risk of new-onset CKD in elderly patients with diabetes was 347.4 μmol/L. Elevated SUA levels were an independent predictor of CKD development in older individuals with diabetes. Diabetes with hyperuricemia was associated with a 1.778-fold higher risk of new-onset CKD than diabetes without hyperuricemia. The correlation was relatively stronger in males than in females. This suggests that stringent SUA control may benefit elderly patients with diabetes; however, whether it is possible to prevent CKD by treating hyperuricemia should be determined by further research.

## Supplementary Material

Supplemental Material

## Data Availability

The data supporting the findings of this study are available upon request from the corresponding author. The data were not publicly available because of privacy or ethical restrictions.
