# Supplementary material for: Serum uric acid is associated with chronic kidney disease in elderly Chinese patients with diabetes
Source: Ren Fail. 2023 Jul 24;45(1):2238825. doi: 10.1080/0886022X.2023.2238825 (PMC10599260; doi:10.1080/0886022X.2023.2238825)
Supplement: Supplemental Material [file IRNF_A_2238825_SM5754.pdf]

Table S1 Characteristics of the diabetes according to the presence of hyperuricemia before and after 1:1 propensity score matching (PSM)

Characteristic	Before 1:1 PSM			After 1:1 PSM		
	Without hyperuricemia (N=25554)	Hyperuricemia (N=13485)	SMD	Without hyperuricemia (N=13485)	Hyperuricemia (N=13485)	SMD
Age	71.83±5.03	72.07±5.19	0.047	72.08±5.09	72.07±5.19	0.002
Sex (female n, %)	16617 (65.03%)	6271 (46.50%)	0.380	6445(47.79%)	6271 (46.50%)	0.026
Diabetic duration	5.0(0-10.0)	4.0(0-10.0)	0.063	4(0-10.0)	4.0(0-10.0)	0.020
BMI (kg/m^2)	24.41±3.23	25.40±3.21	0.308	25.26±3.20	25.40±3.21	0.045
Obesity (n, %)	13531 (52.95%)	8857(65.68%)	0.261	8724(64.69%)	8857(65.68%)	0.021
Hypertension (n, %)	19485 (76.25%)	10967 (81.33%)	0.124	10900(80.83%)	10967 (81.33%)	0.013
SBP (n, %)	12351(48.33%)	6566(48.69%)	0.007	6660(49.39%)	6566(48.69%)	0.005
DBP (n, %)	4834(18.92%)	2753(20.42%)	0.038	2755(20.43%)	2753(20.42%)	<0.001
Smoking (n, %)	2788(10.91%)	1948 (14.45%)	0.106	1950(14.46%)	1948 (14.45%)	<0.001
Drinking (n, %)	2861 (11.20%)	2479 (18.38%)	0.204	2306(17.10%)	2479 (18.38%)	0.034
Dyslipidemia (n, %)	8032 (31.43%)	5666(42.02%)	0.221	5359(39.74%)	5666(42.02%)	0.046
TGs (n, %)	3589(14.04%)	3150(23.36%)	0.241	2821(20.92%)	3150(23.36%)	0.059
TC (n, %)	2356(9.22%)	1294(9.60)	0.013	1272(9.43%)	1294(9.60)	0.006
HDL-C (n, %)	3401(13.31%)	2670(19.33%)	0.164	2467(18.29%)	2670(19.33%)	0.027
LDL-C (n, %)	1385(5.42%)	720(5.34%)	0.004	705(5.23%)	720(5.34%)	0.005
FPG (n, %)	15113(59.14%)	7542(55.93%)	0.065	7619(56.50%)	7542(55.93%)	0.012

Data are shown as mean ± standard deviation for normally distributed variables, median (interquartile range) for non-normally distributed variables, or percentages for categorical variables.

SMD: Standardized Mean Difference; BMI, body mass index; SBP: Systolic blood pressure; DBP: Diastolic blood pressure; TGs: Triglyceride; TC: Total cholesterol; HDL-C, high-density lipoprotein cholesterol; LDL-C, low-density lipoprotein cholesterol; FPG, fasting plasma glucose.

SBP was defined as SBP≥ 140 mmHg. DBP was defined as DBP ≥ 90 mmHg. TGs was defined as TGs ≥ 2.3 mmol/L. TC was defined as TC ≥ 6.2 mmol/L. HDL-C was defined as HDL-C ≤ 1.0 mmol/L. The LDL-C level was defined as LDL-C ≥ 4.1 mmol/L. FPG was defined as FPG ≥7.0 mmol/L

Information Classification: General
